# Health research priorities in Pakistan: A Child Health and Nutrition Research Initiative (CHNRI) exercise

**DOI:** 10.7189/jogh.14.04116

**Published:** 2024-08-23

**Authors:** Seyed Abbas Motevalian, Muhammad Naveed Asghar, Sabeen Afzal, Ejaz Ahmad Khan, Amna Rehana Siddiqui, Tazeen Saeed Ali, Hassanali Dalvi Shirazi

**Affiliations:** 1 Iran University of Medical Sciences, Psychosocial Health Research Institute, Tehran, Iran; 2 Consultant for World Health Organization, Pakistan Country Office, Pakistan; 3 World Health Organization, Pakistan Country Office, Health Systems Development, Islamabad, Pakistan; 4 Ministry of National Health Services, Regulation and Coordination, Islamabad, Pakistan; 5 Health Services Academy, Islamabad, Pakistan; 6 Aga Khan University, Department of Community Health Sciences, Karachi, Pakistan; 7 Aga Khan University, School of Nursing and Midwifery, Karachi, Pakistan

## Abstract

**Background:**

The National Health Vision of Pakistan 2016–2025 is to provide affordable health services through a resilient and responsive health system for achieving health-related Sustainable Development Goals (SDGs) and universal health coverage (UHC) targets. Through this study, we wanted to identify the research priorities at the national level that would help to provide the necessary evidence for achieving this vision through essential package of health services (EPHS).

**Methods:**

We followed World Health Organization (WHO) guidance for undertaking research priority-setting exercises and the Child Health and Nutrition Research Initiative (CHNRI) methodology in conducting this national exercise. The proposed research options for the next three to five years were identified in five thematic research areas: communicable diseases; non-communicable diseases (NCDs) and injuries; reproductive, maternal, newborn, child, and adolescent health (RMNCAH); mental health; and health systems and services. We assessed these research options against five priority-setting criteria: feasibility, public health importance, sustainability, equity, and community involvement.

**Results:**

Forty-three experts proposed 272 research ideas, which were consolidated into a list of 155 research options and sent back to the experts for scoring. The top 10 research priorities in each of the five research areas were identified based on the weighted research priority scores (wRPS) rankings. Tuberculosis and antimicrobial resistance; NCD screening and prevention; maternal and neonatal mortality; mental health of children, adolescents, and youth; and human resource management were the issues that were most frequently reflected in the list of research priorities. Most research priorities aimed to identify barriers to the implementation of interventions.

**Conclusions:**

Through this exercise, we identified the top 50 national health research priorities, which also have a strong correlation with EPHS interventions. To realise the findings of this exercise, funding agencies should establish funding mechanisms to support the undertaking of the identified research priorities, and academic researchers should actually utilise them in future studies. Such activities could hopefully contribute to achieving the national health goals.

The National Health Vision of Pakistan 2016-2025 lays the groundwork for achieving health-related Sustainable Development Goal (SDG) and Universal Health Coverage (UHC) targets [1]. It emphasises the need for national health research that is relevant to the local context and of high quality, transitioning from a focus on medical research to prioritising national health research areas in line with local needs. This shift is crucial for informed policy-making and evidence-based decision-making, as echoed in Pakistan's strategic documents such as the 12th Five Year Plan [2] and the National Action Plan 2019-2023 [3].

To achieve its health coverage goals, Pakistan formulated the National Essential Package of Health Services (EPHS)/UHC benefit package. This initiative was meticulously designed to reflect local health care needs and challenges, while simultaneously incorporating the interventiosn recommended within the globally recognised Disease Control Priorities 3 (DCP3) project. The EPHS encompasses 151 prioritised health interventions dispersed across four clusters: communicable diseases; non-communicable diseases (NCDs) and injuries; reproductive, maternal, newborn, child, adolescent health and nutrition (RMNCAH); and health services [4]. Of these interventions, 117 were designated for district-level implementation, 22 for tertiary-level hospitals, and 12 at the population level. Enhancing evidence-informed policymaking and conducting quality local research are pivotal in progressing towards these visionary goals and targets.

Recent findings from a study on health research funding and output in Pakistan [5] showed that three major local funding agencies – the Higher Education Commission, the Pakistan Science Foundation, and the Pakistan Health Research Council – along with research institutions and international donors, invested about USD 8.5 million over the five fiscal years from 2013 to 2018. This investment was closely linked to research output, highlighting the need for increased funding to enhance health services. However, challenges such as low government prioritisation, inadequate funding, the lack of a national research priority list, and a fragmented funding system present significant obstacles to advancing health research in Pakistan.

Health research priority-setting, a key pillar of the strategy for health research proposed by the World Health Organization (WHO), is vital in this context. In line with its National Health Vision and recent WHO guidance [6], Pakistan has undertaken a national health research priority-setting exercise, becoming one of the first countries in the Eastern Mediterranean Region to do so. Through this exercise, which is endorsed by the WHO and the Ministry of National Health Services Regulation and Coordination, we aimed to identify research priorities essential for achieving the National Health Vision through the implementation of the UHC benefit package EPHS. Well-sourced national health research priorities that emerge from this exercies will significantly contribute to the enhancement of Pakistan's health research agenda and the effectiveness of evidence-informed policy-making.

## METHODS

The Pakistan national health research priority-setting exercise, which was conducted from December 2020 to November 2021, followed the recently-published WHO guidebook for delivering such exercises [6]. First, a steering committee comprising members from the Ministry of National Health Services Regulation and Coordination, WHO country and regional offices, and other key stakeholders was formed to oversee the exercise. The committee established a technical working group (TWG) to lead the priority-setting. It included members of the Ministry of National Health Services Regulation and Coordination, the Higher Education Commission, Public Health Research Council, Health Services Academy, Aga Khan University, Pakistan Medical and Nursing Council, Pakistan Health Council, WHO regional, country offices, and provincial health departments [7].

The TWG outlined the plan for the exercise, focussing on objectives, the timeframe, the target population, research areas, and methodologies. They selected the Child Health and Nutrition Research Initiative (CHNRI) methodology [8] as an appropriate approach. The exercise aimed at prioritising research for the next 3–5 years in five thematic areas in line with the Pakistan EPHS: communicable diseases; NCDs and injuries; RMNCAH; mental health; and health systems and services[7].

We adapted the key steps of the CHNRI method in designing the exercise: forming a management team; selecting criteria for assessing research options; preparing and scoring a long list of research options based on these criteria; and calculating priority scores and ranking research options [8]. The five criteria for assessing research options included feasibility, public health importance, sustainability, equity, and community involvement (**Box 1**).

Box 1 **The priority-setting criteria defining as yes/no questions for Pakistan national health research prioritization exercise**

**Criterion 1: feasibility**
1. Does the capacity of doing this research in Pakistan e.g. technical (human), financial, logistic (infrastructure, equipment, and institutional resources) exist or could be readily provided?
2. Is it likely that this research will be conducted in the next three to five years in terms of obtaining ethical clearance and research funding?

**Criterion 2: public health importance**
1. Does this research address a high burden health problem or risk factors in Pakistan?
2. Does this research focus on the evidence gaps for a national health plan and priorities (based upon needs assessment, measuring service coverage, evaluation of the implementation of health programmes, etc.)?

**Criterion 3: sustanibility**
1. Would the expected outcome(s)/impact of health interventions based on this research be sustainable within the context, especially after funding cessation?
2. Would the expected outcomes of this research be potentially translated into health policy and practice for public health benefit in Pakistan?

**Criterion 4: equity**
1. Is it likely that underprivileged communities would benefit more from the outcomes of this research, hereafter reducing the possible inequities in Pakistan?
2. Does this research focus on health-related issues that mainly affect the underprivileged sectors of the population?

**Criterion 5: community involvement**
1. Is it expected that the community involvement will be beneficial for the outcome of research as an end-user of that particular community?
2. Is this research area ethically acceptable to the community?

We then invited experts to propose research options. Specifically, we identified possible participants based on their publication records [9] and committee members' personal knowledge of these individuals. Forty-three out of approximately 100 invitees agreed to participate and suggest research options related to gaps in national health programmes in the five thematic areas. We provided them with a template with some examples to help them in proposing research options. After the experts submitted the research options, three members of the steering committee refined them and consolidated them into a list, removing duplicates, merging similar items, changing research questions to research statements, and deleting general topics (e.g. ‘maternal, neonatal, and child mortality and morbidity’). We then sent the compiled list back to the researchers and experts whom we asked to score each research option against each of the predefined criteria. They could assign ‘2’ if they agreed that an option met the criterion, ‘0’ if they disagreed, and if they ‘1’ neither neither agreed nor disagreed [10]. They could also leave an option unscored if they had insufficient information. To align with standard CHNRI methodology for comparability, we converted these scores to a 0–1 range and presented them here as such.

We then held a key workshop in March 2021 involving 26 in-person and 43 virtual participants (researchers, experts, federal and provincial health authorities, and funding agency representatives) to further refine and score the options [11]. As an output of their discussions, the stakeholder group established criteria weights, calculated based on a total of 100 weights as follows: feasibility = 24.6, public health importance = 32.8, sustainability = 22.9, equity = 8.2, and community involvement = 11.5.

We computed the weighted research priority scores (wRPS) for each research option, which we then ranked based on their wRPS for each of the five research areas. For each criterion on each research option, we calculated the proportion of the number of scorers who gave the most common score (mode of scores) divided by the total number of scorers (without blank responses) for that item. We then averaged this proportion to obtain the average expert agreement (AEA).

The TWG finalised the ranked list of priorities in subsequent meetings. This included endorsing workshop outcomes; agreeing on criteria weights; and planning for publication, dissemination, and evaluation of the exercise. The research options were further adjusted based on feedback from these activities, ensuring clarity and relevance.

## RESULTS

Forty-three experts proposed 272 research ideas. Three members of the steering committee (SAM, AM, and AA) refined the long list of proposed research options. After removing duplicates and merging similar research options, we compiled a list of 155 research options for voting and scoring, distributed among the five thematic groups as follows: communicable diseases = 34, NCDs = 28, RMNCAH = 36, mental health = 24, and health systems and services = 33. The experts scored the research options against the predefined criteria, while the stakeholders defined the criteria weights. After computing the wRPS, we ranked the top 10 research options in each of the five areas. We classified the research priorities into five research cycle types: problems (describing the burden of a health issue), causes (identifying risk factors and determinants of a health issue), solutions (new interventions, including new policies), implementation (understanding the barriers to implementation of known interventions), and evaluation (evaluating the impact of interventions). The list of the 151 EPHS interventions related to the priroities and their respective codes are presented in Table S1 in the **Online Supplementary Document**.

### Research priorities for communicable diseases

Of the top 10 research priorities in communicable diseases, three were related to tuberculosis (TB), three to antimicrobial resistance (AMR), and two to HIV/AIDS. Other research priorities were related to outbreak preparedness, national immunisation programmes, and hepatitis B vaccination. We observed a higher agreement among the experts on the research priorites related to TB and AMR compared to those related to HIV and vaccination (**Table 1**). Interestingly, none of the three research options (ROs) related to the coronavirus disease 2019 (COVID-19) pandemic or three related to vector-borne diseases ranked among the top 10 research priorities (RPs) in that research domain.

**Table 1:** The top 10 research priorities in communicable diseases, ranked by wRPS

Rank	Research priority	Type of research	Feasibility	Public health importance	Sustainability	Equity	Community involvement	wRPS	AEA	EPHS interventions linked with research priorities
1	Developing local guidelines and systems to strengthen preparedness for and response to disease outbreaks	I	0.97	0.94	0.90	0.90	0.87	0.94	0.84	C46, P8
2	Evaluation of multidrug resistance TB control program	E	0.94	0.97	0.84	0.87	0.90	0.93	0.85	HC27, FLH17, RH2
3	Practical challenges and solutions to TB control in Pakistan	I	0.90	0.94	0.90	0.90	0.84	0.92	0.81	C32, HC28, P5, HC26, HC27, FLH17
4	Developing and evaluating interventions to control AMR	I, S	0.94	0.97	0.82	0.82	0.75	0.90	0.74	RH3, FLH18
5	Strengthening TB case detection at primary health care level (e.g. via community and Lady Health Worker engagement; PHC staff training)	I	0.93	0.90	0.86	0.82	0.86	0.90	0.73	C32, HC26, HC28, P5
6	Multi-drug resistant Typhoid Fever: determinants and interventions	I, C	0.91	0.94	0.85	0.72	0.85	0.90	0.73	None
7	Evaluation of HBV vaccination programme in Pakistan	E	0.90	0.90	0.77	0.74	0.80	0.86	0.69	HC24
8	Empowering and assessing community engagement for trust-building in immunisation activities	I	0.84	0.87	0.77	0.80	0.87	0.85	0.65	C11, C12, C16
9	Facilitators and barriers of harm reduction strategies (needle syringe programmes and opioid substitution therapy) for preventing HIV and hepatitis C transmission in people who inject drugs	I	0.87	0.90	0.77	0.70	0.77	0.85	0.61	C30a, C30b
10	Facilitators and barriers to continuity of care among HIV positives	I	0.80	0.87	0.77	0.77	0.74	0.82	0.71	C28, HC19, HC23

AEA – average expert agreement, AMR – antimicrobial resistance, C – cause, E – evaluation, EPHS – essential package of health services, I – implementation, S – solution, TB – tuberculosis, wRPS –weighted research priority scores

### Research priorities for non-communicable diseases and injuries

Three out of the top ten research priorities in the NCD research domain dealt with NCD screening programmes. Three research priorities were related to cancer (screening, delay in diagnosis, and establishment of a cancer registry); four were focussed on the strengthening of current NCD programmes and interventions. Two research priorities dealt with injuries (evaluation of pre-hospital and hospital care, and child injury prevention). One was focussed on the effects of the COVID-19 pandemic on NCD services (**Table 2**).

**Table 2:** Top 10 research priorities in NCDs and injuries, ranked by wRPS

Rank	Research priority	Type of research	Feasibility	Public health importance	Sustainability	Equity	Community involvement	wRPS	AEA	EPHS interventions linked with research priorities
1	Developing and evaluating interventions to improve access to NCD screening, diagnosis, and management services	I, S	1.00	1.00	0.82	0.94	1.00	0.97	0.90	HC36, HC45, FLH20, FLH22, FLH23, RH4, RH6
2	Strengthening screening programmes for early detection of common cancers in Pakistan (breast cancer, cervical cancer, etc.)	I	0.89	1.00	0.95	0.95	0.89	0.96	0.78	FLH13, RH7, RH8
3	Breast cancer in Pakistan: screening and opportunities for improving survival	I	0.89	1.00	0.89	0.95	0.95	0.95	0.87	RH7
4	Designing, implementing, and evaluating interventions to improve lifestyle modification, prevention, early detection, and management of diabetes mellitus and hypertension	I, S	0.84	0.95	0.67	0.78	0.89	0.85	0.71	P2
5	Establishment of national population-based cancer registry of Pakistan linked with sub-national cancer registries	P, C	0.78	0.95	0.84	0.72	0.61	0.84	0.64	None
6	Evaluating the impact of community health worker programs on NCD prevention and control	E	0.82	0.82	0.75	0.82	1.00	0.84	0.75	None
7	Evaluation of pre-hospital (e.g.: 1122) and hospital care of injuries in Pakistan	E	0.84	0.89	0.72	0.67	0.78	0.82	0.60	HC60, HC61, HC62, HC64, FLH30, FLH33, FLH36, FLH39, FLH41b, FLH45, FLH48a, FLH48b, FLH49, FLH50, FLH52, RH10, RH11
8	Determinants and distribution of delayed diagnosis and management of cancers and hematological malignancies in Pakistan	P, C, I	0.84	0.84	0.72	0.78	0.78	0.81	0.62	FLH13, RH7, RH8, RH9
9	Prevention and management of NCDs during COVID-19 pandemic	I	0.75	0.82	0.75	0.75	0.75	0.79	0.58	None
10	Determinants and interventions to present at the household level to reduce the risk of injuries among children	I, C	0.67	0.89	0.67	0.72	0.84	0.78	0.62	None

AEA – average expert agreement, C – cause, E – evaluation, EPHS – essential package of health services, I – implementation, NCD – non-communicable disease, S – solution, wRPS –weighted research priority scores

None of the four proposed ROs were related to genetic disorders or genetic susceptibility to NCDs, three ROs focussed on road traffic injuries, and two ROs on tobacco smoking received enough votes to be ranked among the top 10 priorities.

### Research priorities for RMNCAH

Three research priorities within this domain focussed on maternal mortality. Three of the top priorities were linked with nutrition, child growth, and development. Neonatal mortality and family planning were also placed within one research priority, each (**Table 3**). Most of the non-priority ROs in the RMNCAH category were connected with reducing maternal and neonatal mortality and improving child nutrition.

**Table 3:** Top 10 research priorities in RMNCAH, ranked by wRPS

Rank	Research priority	Type of research	Feasibility	Public health importance	Sustainability	Equity	Community involvement	wRPS	AEA	EPHS interventions linked with research priorities
1	Designing and assessing interventions to improve the quality of care provided to pregnant women at all levels (including preconception, antenatal, and obstetric care)	I, S	1.00	1.00	0.80	0.95	1.00	0.97	0.92	C3a, HC9a, C3c, C5, HC2, HC3, HC7, HC9b, HC11, FLH4, FLH5, FLH7, FLH8, FLH10, HC10
2	Developing, implementing, and assessing interventions to reduce stunting in children less than five years of age	I, S	0.90	0.95	0.90	0.85	0.85	0.92	0.78	C8, C14, FLH12
3	Identifying determinants of high neonatal mortality and effective interventions to reduce it (to meet SDGs 2030)	C, I	0.90	1.00	0.80	0.90	0.85	0.92	0.78	C2, C3b, C3d, HC1, HC5a, HC5b, HC6, FLH1, FLH3, FLH6, FLH11
4	Sustainable interventions to improve maternal nutrition status during pregnancy (to improve maternal and foetal health)	I	0.90	0.95	0.80	0.90	0.95	0.92	0.82	C27a, C27b
5	Developing and evaluating interventions to improve the provision of and access to community family planning and post-abortion care services (e.g. EmONC)	I, S	0.85	0.95	0.85	0.85	0.95	0.91	0.80	C1, HC2, HC11
6	Developing, utilising, and assessing m-health interventions to enhance awareness among mothers (for growth and development of newborns)	S, I	0.95	0.95	0.65	0.85	0.80	0.87	0.76	None
7	Best mechanisms for the promotion of family planning services (e.g. training of health care workers, supplies management at public and private facilities)	I, S	0.90	0.85	0.80	0.90	0.85	0.87	0.76	C1
8	Developing and assessing interventions to address social determinants of maternal mortality and poor MNCH care (e.g. stigmatization, marital disharmony, domestic violence, cast, religion, teenage pregnancy, cultural practices)	I, S	0.85	0.95	0.75	0.90	0.75	0.87	0.58	C25
9	Assessing the capacity of neonatal care and development (personnel: neonatologists, midwives, outreach workers; equipment, etc.)	P, I	0.80	1.00	0.80	0.75	0.70	0.87	0.66	C2, C3b, C3d, HC1, HC5a, HC5b, FLH6, HC6
10	Maternal mortality in Pakistan: determinants, interventions and their assessment	P, C, I	0.94	0.89	0.67	0.78	0.89	0.86	0.72	C3a, HC9a, C3c, C5, HC2, HC3, HC7, HC9b, HC11, FLH4, FLH5, FLH7, FLH8, FLH10, FLH38, HC10

AEA – average expert agreement, C – Cause, E – evaluation, EmONC – emergency obstetric and ewborn care, EPHS – essential package of health services, I – implementation, MNCH – maternal, newborn, and child health, S – solution, SDG – Sustainable Development Goal, wRPS –weighted research priority scores

### Research priorities for mental health

Experts were more concerned about the mental health of children, adolescents, and youth, as their scores had pushed five ROs of this age group to the top ten priorities. Women’s mental health came up as another important issue linked to two RPs (**Table 4**). Gender violence and substance abuse were the two most frequent issues among the non-priority research options.

**Table 4:** Top 10 research priorities in mental health, ranked by wRPS

Rank	Research priority	Type of research	Feasibility	Public health importance	Sustainability	Equity	Community involvement	wRPS	AEA	EPHS interventions linked with research priorities
1	Developing and assessing interventions to improve early detection and management of common mental health problems (including anxiety and depression) in Pakistan	I	1.00	0.93	0.93	0.79	0.93	0.95	0.86	HC50
2	Knowledge, attitudes, and practices study on community regarding child abuse and neglect (risk factors and consequences)	P, C	0.93	0.93	0.93	0.93	0.93	0.95	0.86	None
3	Adolescent mental health and behaviour issues: situation analysis, challenges, and proposed solutions	P, C, I	0.93	0.93	0.86	0.86	0.93	0.92	0.77	HC14, P1
4	Developing and assessing strategies for early detection and management of adolescent mental health problems in school settings (for teachers and counselors)	I, S	0.93	0.93	0.86	0.86	0.93	0.92	0.83	None
5	Epidemiology of youth suicide and suicidal behaviour	P, C	0.93	0.93	0.93	0.79	0.75	0.91	0.80	None
6	Developing and assessing school-based interventions effective in building mental health resilience in children and young adults	I, S	0.86	0.93	0.72	0.79	0.93	0.87	0.74	None
7	Epidemiology and prevention of dementia in Pakistan	P, C, I	0.79	0.93	0.79	0.86	0.72	0.85	0.66	None
8	Workplace harassment among female workers working in Pakistan: epidemiology and preventive interventions	P, C, I	0.93	0.72	0.93	0.86	0.72	0.84	0.71	HC16
9	Promoting mental well-being and help-seeking behaviour among the population at risk	I, S	0.72	0.93	0.79	0.79	0.72	0.82	0.63	None
10	Epidemiology and prevention of post-partum depression in Pakistani communities	P, C, I	0.79	0.86	0.79	0.79	0.72	0.82	0.63	None

AEA – average expert agreement, C – cause, E – evaluation, EPHS – essential package of health services, I – implementation, S – solution, wRPS –weighted research priority scores

### Research priorities for health systems and services

Human resources came up as an issue of concern for our experts, as they were the subject of seven of the top 10 priorities in this category (**Table 5**). Interestingly, 11 out of 23 non-priority ROs in this category were also connected with human resources and capacity building.

**Table 5:** Top 10 research priorities in health systems and services, ranked by wRPS

Rank	Research priority	Type of research	Feasibility	Public health importance	Sustainability	Equity	Community involvement	wRPS	AEA	EPHS interventions linked with research priorities
1	Upgrading national health information systems and health workforce in Pakistan (technologically, quality, etc.)	I	1.00	1.00	0.80	0.70	0.65	0.91	0.72	None
2	Needs assessment, strengthening, and evaluating contributions of Lady Health Workers to health care (e.g. for tuberculosis referrals/contact tracing; immunisation and maternal and child health services)	E, I	0.85	0.95	0.80	0.85	0.90	0.89	0.72	C4
3	Supply vs demand factors/barriers for effective health service delivery in rural settings	I	0.95	1.00	0.65	0.89	0.75	0.89	0.74	None
4	Mechanisms for retention of primary health care personnel (including training, incentives, etc.)	I, S	0.95	0.95	0.75	0.80	0.70	0.88	0.76	None
5	Mechanisms for integration and capacity building of traditional and non traditional healers in rural health care systems (e.g. midwives, Lady Health Visitors, Lady Health Workers, nurse practitioners)	I, S	0.85	0.80	0.85	0.85	0.90	0.86	0.66	None
6	Strategies to expand the nursing workforce (capacity building, incentives, etc.)	I, S	0.85	0.90	0.85	0.72	0.65	0.85	0.68	None
7	Implementation strategy for equipment inventory system	I	0.90	0.85	0.85	0.67	0.69	0.84	0.66	None
8	Interventions for health care delivery by Lady Health Workers during the COVID-19 pandemic	I, S	0.80	0.85	0.75	0.75	0.80	0.82	0.7	None
9	Addressing cost containment via innovative technologies (e.g. laboratory information systems, health management information systems, and telemedicine)	I, S	0.85	0.75	0.85	0.70	0.70	0.80	0.58	RH5
10	Interventions to improve continuous educations for health professionals (e.g. continuing medical education)	I, S	0.80	0.80	0.85	0.65	0.65	0.80	0.64	None

AEA – average expert agreement, C – cause, E – evaluation, EPHS – essential package of health services, I – implementation, S – solution, wRPS –weighted research priority scores

### Types of research priorities and their link with EPHS interventions

Most of the research priorities (64.0%) could be answered by more than one type of research. The majority of research priorities (86.0%) dealt with barriers to implementation. In the mental health research domain, 60.0% of the research priorities had both ‘problem’ and ‘cause’ research type components (**Table 6**).

**Table 6:** Research priorities in five research areas, grouped by research cycle types

Research type	Communicable diseases	NCDs	RMNCAH	Mental health	Health systems and services
Problem	0	2	2	6	0
Cause	1	3	2	6	0
Solution	1	2	6	3	6
Implementation	8	7	10	8	10
Evaluation	2	2	0	0	1
More than one	2	5	9	9	7

NCD – non-communicable diseases, RMNCAH – reproductive, maternal, newborn, child, and adolescent health

The identified research priorities were strongly correlated with EPHS interventions. Out of 151 health interventions, 90 were linked with at least one research priority, while out of 50 research priorities, 29 were linked with at least one health intervention. Some of the EPHS interventions were linked with multiple RPs, while 40% of them were not linked with any research priority (Table S1 in the **Online Supplementary Document**).

## DISCUSSION

This is the first national health research priority-setting exercise to be delivered in Pakistan; it has resulted in a list of 50 research priorities dispersed in the five main research areas, which are, in turn, in line with the four clusters of Pakistan’s EPHS. A review of the literature showed that, although there have been efforts to identify research priorities at the national level in Pakistan [12,13], none had published a list of research priorities. Our exercise was also a pilot for applying the WHO guidance on undertaking research priority-setting exercises. The WHO guidance [6] reviewed the five common research priority-setting methods, including the CHNRI, which is described as a comprehensive metric-based method that follows the ‘wisdom of the crowd’ approach. This priority-setting exercise inclusively engaged researchers from universities and research institutions; policymakers from national and provincial health departments; funding agencies; and other stakeholders, ranging from professional associations and international organisations. As shown previously [14], we found the CHNRI to be a systematic, transparent, inclusive, and particularly flexible research priority-setting process.

Exploring the linkage between research priorities and the EPHS interventions, we observed that about 60% of the interventions are linked with at least one research priority. The 61 EPHS interventions that had no link with research priorities were mostly focussed on tropical diseases, dental care, visual impairments, rehabilitation, and medical or surgical interventions at first-level or referral hospitals. Conversely, 29 (58%) of research priorities were linked with at least one of the EPHS interventions. Twenty-one research priorities not linked with EPHS interventions were mostly related to the health workforce, health information systems, violence and injury prevention, and improvement of mental health services.

The issues that were more concerning for the exercise participants and received higher scores were TB, AMR, and HIV/AIDS (communicable diseases research area); screening and prevention of NCDs and cancer diagnosis (NCD research area); maternal and neonatal mortality, child nutrition and growth, and family planning (RMNCAH research area); mental health of children, adolescents, and youth (mental health research area); and human resource management (health systems and services research area). Almost all (n/N = 48/50) of the research priorities received wRPSs of 0.8 or higher. In all of the five research areas, the first-ranked research priority that usually received the highest AEA was a broad topic of research rather than a specific research question. As we anticipated, the research options that were focussed on an important problem and were wide enough to include the study of distribution, determinants, and an assessment of new interventions were more attractive and more likely to receive a consensus vote. The lower rank research priorities tended to be narrower and were more specific research questions.

The list of research priorities shows that implementation research dominated this exercise. We can think of several reasons for this tendency. The first is the short time frame (three to five years) of this exercise that inevitably favours implementation research, as it is more likely to lead to improvements in population health in a short-term period. The second reason is that most of the research priorities were related to known health issues, with similarly known disease burden, determinants, and effective interventions; therefore, the main research questions were more likely to focus on the barriers to the implementation of interventions. The third reason is the purpose of this exercise, which was to provide evidence for achieving the goals of national health programme; in this situation, we might have expected that implementation and operation research would be prioritised. The predominance of research priorities with ‘problem’ and ‘cause’ research type in the field of mental health shows that this research area is less known than the other four fields.

The Global Burden of Disease (GBD) 2019 study estimates showed that neonatal disorders were the first cause of the burden of disease in Pakistan, and were responsible for 24.2% of total disability-adjusted life years (DALYs) lost. Ischaemic heart diseases, lower respiratory tract infections, diarrhoeal diseases, tuberculosis, stroke, congenital birth defects, and diabetes mellitus were the 2nd through 8th causes of burden of diseases. These causes together are responsible for 26.3% of total lost DALYs [15]. The importance of neonatal mortality, TB, and NCD screening and prevention is reflected in the list of national research priorities. Yet despite the high burden and importance of lower respiratory infections, diarrheal diseases, and COVID-19 in Pakistan, they are not reflected enough among the research priorities in this exercise. We hope that they will receive more attention in future revisions of the national research priorities.

The health research priority-setting exercise in Pakistan, endorsed by WHO and the Ministry of National Health Services Regulation and Coordination, represents a significant step towards aligning research with national health goals. The use of the CHNRI methodology, supported by a diverse steering committee and TWG, underscores the exercise's methodological rigour and comprehensive stakeholder engagement. This approach ensured a wide range of perspectives in identifying priorities, enhancing the relevance and applicability of the findings to Pakistan's health system needs.

However, this study has some inherent limitations. The selection of experts, primarily based on publication records and committee members' knowledge, might have introduced selection bias, potentially limiting the diversity of research options considered. Additionally, despite being systematic, the subjective nature of the scoring process could reflect individual biases or knowledge gaps among experts. Moreover, the combination of in-person and virtual participation in workshops might have impacted the depth of discussions and consensus-building, potentially affecting the prioritisation outcomes. These insights highlight the need for careful consideration of expert selection and scoring processes in future research prioritisation exercises. Ensuring a broad and inclusive approach to participant selection and fostering equitable engagement among all participants will be crucial in enhancing the robustness and inclusivity of such efforts.

The implementation, publishing and dissemination, and evaluation plans of the Pakistan research priority-setting exercise were discussed in exercise meetings and endorsed by the TGW. They suggested that the international and national research funding agencies (like the Higher Education Commission), in collaboration with the Ministry of National Health Services, Regulations, and Coordination, may establish a funding mechanism (e.g. a call for proposal) to support the implementation of the 50 national health research priorities. By publishing and disseminating research priorities, universities and research institutions will have the opportunity to conduct studies related to the prioritised research options and providing evidence for achieving national health goals.

## CONCLUSIONS

The national health research priority-setting exercise in Pakistan is a significant step in aligning research with the country's health goals, as it successfully identified 50 key priorities with input from a diverse range of stakeholders. Using the CHNRI method, we meticulously planned the exercise and engaged a wide array of health care stakeholders, ensuring that the selected priorities were both highly relevant and readily applicable to Pakistan's health care needs. One key achievement of our work is the effective linkage of most of said research priorities with the EPHS; this ensured that the identified priorities would directly contribute to improving health services and outcomes in Pakistan. The proposed establishment of a funding mechanism by national and international agencies represents a crucial move towards realising these priorities, providing a clear path for academic and research institutions to make significant contributions.

## Additional material


Online Supplementary Document
