# Supplementary material for: Health research priorities in Pakistan: A Child Health and Nutrition Research Initiative (CHNRI) exercise
Source: J Glob Health. 2024 Aug 23;14:04116. doi: 10.7189/jogh.14.04116 (PMC11341109; doi:10.7189/jogh.14.04116)
Supplement: Online Supplementary Document [file jogh-14-04116-s001.pdf]

Table S1- EPHS interventions linked with 50 research priorities

No.	Level	Platform	Cluster	Package	DCP Code	Intervention Name	RP
1	District	Community	RMNCH	Maternal and new-born health	C1	Antenatal and postpartum education on family planning	RMNCAH5, RMNCAH7
2	District	Community	RMNCH	Maternal and new-born health	C2	Counselling of mothers on providing thermal care for preterm new-borns (delayed bath and skin to skin contact)	RMNCAH3, RMNCAH9
3	District	Community	RMNCH	Maternal and new-born health	C3a	Management of labour and delivery in low-risk women by skilled attendant	RMNCAH1, RMNCAH10
4	District	Community	RMNCH	Maternal and new-born health	C3b	Basic neonatal resuscitation following delivery	RMNCAH3, RMNCAH9
5	District	Community	RMNCH	Maternal and new-born health	C4	Promotion of breastfeeding or complementary feeding by lay health workers	HSS2
6	District	Community	RMNCH	Child health	C8	Detection and management of severe acute malnutrition and referral in the presence of complications	RMNCAH2
7	District	Community	RMNCH	Child health	C10	Education on handwashing and safe disposal of children's stools	
8	District	Community	RMNCH	Child health	C11	Pneumococcus vaccination	CD8
9	District	Community	RMNCH	Child health	C12	Rotavirus vaccination	CD8
10	District	Community	RMNCH	Child health	C14	Provision of vitamin A and zinc supplementation to children according to WHO guidelines, and provision of food supplementation to women and children in food insecure households	RMNCAH2
11	District	Community	RMNCH	Child health	C16	Childhood vaccination series (diphtheria, pertussis, tetanus, polio, BCG, measles, hepatitis B, HiB, and rubella)	CD8
12	District	Community	RMNCH	School-age health	C18	Education of school children on oral health	
13	District	Community	RMNCH	School-age health	C19	Vision pre-screening by teachers; vision tests and provision of ready-made glasses on-site by eye specialists	
14	District	Community	RMNCH	Reproductive health	C27a	Provision of iron and folic acid supplementation to pregnant women, and provision of food or caloric supplementation to pregnant women in food-insecure households	RMNCAH4
15	District	Community	Infectious diseases	HIV	C28	Community-based HIV testing and counselling (for example, mobile units and venue-based testing), with appropriate referral or linkage to care and immediate initiation of lifelong ART	CD10
16	District	Community	Infectious diseases	HIV	C30a	Provision of condoms	CD9
17	District	Community	Infectious diseases	HIV	C30b	Provision of disposable syringes	CD9
18	District	Community	Infectious diseases	TB	C32	Routine contact tracing to identify individuals exposed to TB and link them to care	CD3, CD5
19	District	Community	Infectious diseases	NTDs	C43	Early detection and treatment of Chagas disease, human African trypanosomiasis, leprosy, and leishmaniases	
20	District	Community	Infectious diseases	Pandemics	C45	Identify and refer patients with high risk including pregnant women, young children, and those with underlying medical conditions	
21	District	Community	Infectious diseases	Pandemics	C46	In the context of an emerging infectious outbreak, provide advice and guidance on how to recognize early symptoms and signs and when to seek medical attention	CD1
22	District	Community	NCIP	Environmental	C51	WASH behaviour change interventions, such as community-led total sanitation	
23	District	Community	Health services	Rehabilitation	C53a	Identification of ECD rehabilitation interventions	
24	District	Community	RMNCH	Maternal and new-born health	HC4a	Condoms and hormonal contraceptives	
25	District	Community	RMNCH	Maternal and new-born health	HC5a	Counselling on kangaroo care for new-borns	RMNCAH3
26	District	Community	RMNCH	Maternal and new-born health	HC9a	Screening of hypertensive disorders in pregnancy	RMNCAH1, RMNCAH10

27	District	Community	Infectious diseases	TB	HC28	Screening for HIV in all individuals with a diagnosis of active TB; if HIV infection is present, start (or refer for) ARV treatment and HIV care	CD3, CD5
28	District	Community	Infectious diseases	TB	P5	Systematic identification of individuals with TB symptoms among high-risk groups and linkage to care ("active case finding")	CD3, CD5
29	District	Health Centre	RMNCH	Maternal and new-born health	C3c	Management of labour and delivery in low-risk women by skilled attendant	RMNCAH1, RMNCAH10
30	District	Health Centre	RMNCH	Maternal and new-born health	C3d	Basic neonatal resuscitation following delivery	RMNCAH3, RMNCAH9
31	District	Health Centre	Infectious diseases	Adult febrile illness	C33	For malaria due to *Plasmodium vivax* , test for G6PD deficiency; if normal, add chloroquine or chloroquine plus 14-day course of primaquine	
32	District	Health Centre	RMNCH	Maternal and new-born health	C5	Tetanus toxoid immunization among schoolchildren and among women attending antenatal care	RMNCAH1, RMNCAH10
33	District	Health Centre	RMNCH	Reproductive health	C27b	Provision of iron and folic acid supplementation to pregnant women, and provision of food or caloric supplementation to pregnant women in food-insecure households	RMNCAH4
34	District	Health Centre	Health services	Rehabilitation	C53b	ECD rehabilitation interventions	
35	District	Health Centre	RMNCH	Maternal and new-born health	HC1	Early detection and treatment of neonatal pneumonia with oral antibiotics	RMNCAH3, RMNCAH9
36	District	Health Centre	RMNCH	Maternal and new-born health	HC2	Management of miscarriage or incomplete abortion and post abortion care	RMNCAH1, RMNCAH5, RMNCAH10
37	District	Health Centre	RMNCH	Maternal and new-born health	HC3	Management of preterm premature rupture of membranes, including administration of antibiotics	RMNCAH1, RMNCAH10
38	District	Health Centre	RMNCH	Maternal and new-born health	HC4b	Condoms and hormonal contraceptives	
39	District	Health Centre	RMNCH	Maternal and new-born health	HC5b	Counselling on kangaroo care for new-borns	RMNCAH3, RMNCAH9
40	District	Health Centre	RMNCH	Maternal and new-born health	HC7	Pharmacological termination of pregnancy	RMNCAH1, RMNCAH10
41	District	Health Centre	RMNCH	Maternal and new-born health	HC9b	Screening and management of hypertensive disorders in pregnancy	RMNCAH1, RMNCAH10
42	District	Health Centre	RMNCH	Maternal and new-born health	HC11	Management of labour and delivery in low-risk women (BEmNOC), including initial treatment of obstetric or delivery complications prior to transfer	RMNCAH1, RMNCAH5, RMNCAH10
43	District	Health Centre	RMNCH	Child health	HC12	Detection and treatment of childhood infections with danger signs (IMCI)	
44	District	Health Centre	RMNCH	Adolescent health	HC14	Psychological treatment for mood, anxiety, ADHD and disruptive behaviour disorders in adolescents	MH3
45	District	Health Centre	RMNCH	Reproductive health	HC16	Post-gender-based violence care, including counselling, provision of emergency contraception, and rape-response referral (medical and judicial)	MH8
46	District	Health Centre	RMNCH	Reproductive health	HC17	Syndromic management of common sexual and reproductive tract infections (for example, urethral discharge, genital ulcer, and others) according to WHO guidelines	
47	District	Health Centre	Infectious diseases	HIV	HC20	Hepatitis B and C testing of individuals identified in the national testing policy (based on endemicity and risk level), with appropriate referral of positive individuals to trained providers	
48	District	Health Centre	Infectious diseases	HIV	HC21	Partner notification and expedited treatment for common STIs, including HIV	
49	District	Health Centre	Infectious diseases	HIV	HC23	Provider-initiated testing and counselling for HIV, STIs, and hepatitis for all in contact with the health system in high prevalence settings, including prenatal care with appropriate referral or linkage to care including immediate ART initiation for those testing positive for HIV	CD10
50	District	Health Centre	Infectious diseases	HIV	HC25	Provision of voluntary medical male circumcision in settings with high prevalence of HIV	

51	District	Health Centre	Infectious diseases	TB	HC26	For PLHIV and children under five who are close contacts or household members of individuals with active TB, perform symptom screening and chest radiograph; if there is no active TB, provide isoniazid preventive therapy according to current WHO guidelines	CD3, CD5
52	District	Health Centre	Infectious diseases	TB	HC27	Diagnosis of TB, including assessment of rifampicin resistance using rapid molecular diagnostics (UltraXpert), and initiation of first-line treatment per current WHO guidelines for drug susceptible TB; referral for confirmation, further assessment of drug resistance, and treatment of drug-resistant TB	CD2, CD3
53	District	Health Centre	Infectious diseases	Adult febrile illness	HC30	Evaluation and management of fever in clinically stable individuals using WHO IMAI guidelines, with referral of unstable individuals to first-level hospital care	
54	District	Health Centre	Infectious diseases	Adult febrile illness	HC32	Provision of insecticide-treated nets to children and pregnant women attending health centres	
55	District	Health Centre	Infectious diseases	Pandemics	HC33	Identify and refer to higher levels of healthcare patients with sings of progressive illness	
56	District	Health Centre	NCDIP	CVD	HC36	Long-term combination therapy for persons with multiple CVD risk factors, including screening for CVD in community settings using non-lab-based tools to assess overall CVD risk	NCD1
57	District	Health Centre	NCIP		HC37	Low-dose inhaled corticosteroids and bronchodilators for asthma and for selected patients with COPD	
58	District	Health Centre	NCIP		HC38	Provision of aspirin for all cases of suspected acute myocardial infarction	
59	District	Health Centre	NCIP		HC39a	Screening and management of albuminuric kidney disease with ACEi or ARBs, including targeted screening among people with diabetes	
60	District	Health Centre	NCIP		HC41	Secondary prophylaxis with penicillin for rheumatic fever or established rheumatic heart disease	
61	District	Health Centre	NCIP		HC42	Treatment of acute pharyngitis in children to prevent rheumatic fever	
62	District	Health Centre	NCIP		HC45	Opportunistic screening for hypertension for all adults and initiation of treatment among individuals with severe hypertension and/or multiple risk factors	NCD1
63	District	Health Centre	NCIP		HC50	Management of depression and anxiety disorders with psychological and generic antidepressant therapy	MH1
64	District	Health Centre	NCIP		HC56	Targeted screening for congenital hearing loss in high-risk children, using otoacoustic emissions testing	
65	District	Health Centre	Health services		HC57a	Dental extraction	
66	District	Health Centre	Health services		HC58a	Drainage of dental abscess	
67	District	Health Centre	Health services		HC59	Drainage of superficial abscess	
68	District	Health Centre	Health services		HC60	Management of non-displaced fractures	NCD7
69	District	Health Centre	Health services		HC61	Resuscitation with basic life support measures	NCD7
70	District	Health Centre	Health services		HC62	Suturing of lacerations	NCD7
71	District	Health Centre	Health services		HC63a	Treatment of caries	
72	District	Health Centre	Health services		HC64	Basic management of musculoskeletal and neurological injuries and disorders, such as prescription of simple exercises and sling or cast provision	NCD7
73	District	Health Centre	Health services		HC68	Health centre pathology services	
74	District	First-level hospital	RMNCH		FLH1	Detection and management of foetal growth restriction	RMNCAH3
75	District	First-level hospital	RMNCH		FLH3	Jaundice Management of Phototherapy	RMNCAH3
76	District	First-level hospital	RMNCH		FLH4	Management of eclampsia with magnesium sulphate, including initial stabilization at health centres	RMNCAH1, RMNCAH10
77	District	First-level hospital	RMNCH		FLH5	Management of maternal sepsis, including early detection at health centres	RMNCAH1, RMNCAH10

78	District	First-level hospital	RMNCH		FLH6	Management of new-born complications, neonatal meningitis, and other very serious infections requiring continuous supportive care (such as IV fluids and oxygen)	RMNCAH3, RMNCAH9
79	District	First-level hospital	RMNCH	Maternal and new-born health	FLH7	Management of preterm labour with corticosteroids, including early detection at health centres	RMNCAH1, RMNCAH10
80	District	First-level hospital	RMNCH	Maternal and new-born health	FLH8	Management of labour and delivery in high-risk women, including operative delivery (CEmONC)	RMNCAH1, RMNCAH10
81	District	First-level hospital	RMNCH	Maternal and new-born health	FLH10	Surgical termination of pregnancy by manual vacuum aspiration and dilation and curettage	RMNCAH1, RMNCAH10
82	District	First-level hospital	RMNCH	Child health	FLH11	Full supportive care for severe childhood infections with danger signs	RMNCAH3
83	District	First-level hospital	RMNCH	Child health	FLH12	Management of severe acute malnutrition associated with serious infections	RMNCAH2
84	District	First-level hospital	RMNCH	Reproductive health	FLH13	Early detection and treatment of early-stage cervical cancer	NCD2, NCD8
85	District	First-level hospital	RMNCH	Reproductive health	FLH14	Insertion and removal of long-lasting contraceptives (IUCDs and implants)	
86	District	First-level hospital	RMNCH	Reproductive health	FLH15	Tubal ligation	
87	District	First-level hospital	RMNCH	Reproductive health	FLH16	Vasectomy	
88	District	First-level hospital	Infectious diseases	TB	FLH17	Referral of cases of treatment failure for drug susceptibility testing; enrolment of those with MDR-TB for treatment per WHO guidelines (either short- or long-term regimen)	CD2, CD3
89	District	First-level hospital	Infectious diseases	Adult febrile illness	FLH18	Evaluation and management of fever in clinically unstable individuals using WHO IMAI guidelines, including empiric parenteral antimicrobials and antimalarial and resuscitative measures for septic shock	CD4
90	District	First-level hospital	NCIP	CVD	FLH20	Management of acute coronary syndromes with aspirin, unfractionated heparin and generic thrombolytic (when indicated)	NCD1
91	District	First-level hospital	NCIP	CVD	FLH22	Management of acute coronary exacerbations of asthma and COPD using systemic steroids, inhaled beta-agonists and if indicated oral antibiotics and oxygen therapy	NCD1
92	District	First-level hospital	NCIP	CVD	FLH23	Medical management of acute heart failure	NCD1
93	District	First-level hospital	NCIP	Cancer	FLH24	Management of bowel obstruction	
94	District	First-level hospital	NCIP	Injury	FLH30	Management of intoxication/ poisoning syndromes using widely available agents eg, charcoal, naloxone, bicarbonate, antivenin	NCD7
95	District	First-level hospital	Health services	Surgery	FLH31	Appendectomy	
96	District	First-level hospital	Health services	Surgery	FLH34	Colostomy (adult and paediatric)	
97	District	First-level hospital	Health services	Surgery	FLH35	Escharotomy or fasciotomy (adults)	
98	District	First-level hospital	Health services	Surgery	FLH36	Fracture reduction	NCD7
99	District	First-level hospital	Health services	Surgery	FLH38	Hysterectomy for uterine rupture or intractable postpartum haemorrhage	RMNCAH10
100	District	First-level hospital	Health services	Surgery	FLH39	Irrigation and debridement of open fracture	NCD7
101	District	First-level hospital	Health services	Surgery	FLH41a	Management of septic arthritis	
102	District	First-level hospital	Health services	Surgery	FLH41b	Placement of external fixation and use of traction for fractures	NCD7
103	District	First-level hospital	Health services	Surgery	FLH42	Relief of urinary obstruction by catheterization for fractures	
104	District	First-level hospital	Health services	Surgery	FLH43	Removal of gallbladder, including emergency surgery	
105	District	First-level hospital	Health services	Surgery	FLH44	Repair of perforations (for example perforated peptic ulcer, typhoid ileal perforation)	
106	District	First-level hospital	Health services	Surgery	FLH45	Resuscitation with advanced life support measures, including surgical airway	NCD7
107	District	First-level hospital	Health services	Surgery	FLH48a	Trauma laparotomy	NCD7

108	District	First-level hospital	Health services	Surgery	FLH49	Trauma related amputations	NCD7
109	District	First-level hospital	Health services	Surgery	FLH50	Tube thoracostomy	NCD7
110	District	First-level hospital	Health services	Rehabilitation	FLH52	Compression therapy for amputations, burns, and vascular or lymphatic disorders	NCD7
111	District	First-level hospital	RMNCH	Maternal and new-born health	HC6	Management of neonatal sepsis, pneumonia, and meningitis using injectable and oral antibiotics	RMNCAH3, RMNCAH9
112	District	First-level hospital	RMNCH	Maternal and new-born health	HC10	Screening and management of diabetes in pregnancy (gestational diabetes or pre-existing type II diabetes)	RMNCAH1, RMNCAH10
113	District	First-level hospital	Infectious diseases	HIV	HC19	For individuals testing positive for hepatitis B and C, assessment of treatment eligibility by trained providers followed by initiation and monitoring of ART when indicated	CD10
114	District	First-level hospital	Infectious diseases	HIV	HC24	Hepatitis B vaccination for high-risk populations, including healthcare workers, IDU, MSM, household contacts and partners with multiple sex partners	CD7
115	District	First-level hospital	Health services	Surgery	HC57b	Dental extraction	
116	District	First-level hospital	RMNCH	Maternal and new-born health	RH1	Full supportive care for preterm new-borns	
117	District	First-level hospital	Health services	Surgery	RH14	Cataract extraction and insertion of intraocular lens	
118	Referral hospital		NCIP	Musculoskeletal	FLH25	Calcium and vitamin D supplementation for secondary prevention of osteoporosis	
119	Referral hospital		Health services	Surgery	FLH37b	Hernia Repair, including emergency surgery for neonates and infants	
120	Referral hospital		Health services	Surgery	FLH40	Management of osteomyelitis, including surgical debridement	
121	Referral hospital		Health services	Surgery	FLH41C	Management of septic arthritis	
122	Referral hospital		Health services	Surgery	FLH48b	Trauma laparotomy in children	NCD7
123	Referral hospital		Health services	Surgery	FLH33	Craniotomy for trauma	NCD7
124	Referral hospital		Infectious diseases	TB	RH2	Specialized TB services, including management of MDR- and XDR-TB treatment failure and surgery for TB	CD2
125	Referral hospital		Infectious diseases	Adult febrile illness	RH3	Management of refractory illness including etiological diagnosis at reference microbial laboratory	CD4
126	Referral hospital		NCIP	CVD	RH4	Management of acute ventilator failure due to acute exacerbations of asthma and COPD	NCD1
127	Referral hospital		NCIP	CVD	RH5	Retinopathy screening via telemedicine, followed by treatment using laser photocoagulation	HSS9
128	Referral hospital		NCIP	CVD	RH6	Use of percutaneous coronary intervention for acute myocardial infarction where resources permit	NCD1
129	Referral hospital		NCIP	Cancer	RH7	Treatment of early-stage breast cancer with appropriate multimodal approaches (including generic chemotherapy) with curative intent for cases detected by clinical examination at health centres and first level hospitals	NCD2, NCD3, NCD8
130	Referral hospital		NCIP	Cancer	RH8	Treatment of early-stage colorectal cancer with appropriate multimodal approaches (including generic chemotherapy) with curative intent for cases detected by clinical examination at health centres and first level hospitals	NCD2, NCD8
131	Referral hospital		NCIP	Cancer	RH9	Treatment of early-stage childhood cancers (such as Burkitt and Hodgkin lymphoma, acute lymphoblastic leukaemia, retinoblastoma and Wilms tumour) with curative intent in paediatric cancer units or hospitals	NCD8
132	Referral hospital		NCIP	Musculoskeletal	RH10	Elective surgical repair of common orthopaedic injuries (for example meniscal and ligamentous tears) in individuals with severe functional limitation	NCD7
133	Referral hospital		NCIP	Musculoskeletal	RH11	Urgent, definitive surgical management of orthopaedic injuries (for example open reduction and internal fixation)	NCD7
134	Referral hospital		NCIP	Congenital disorders	RH12	Repair of cleft lip and cleft palate	

135	Referral hospital	NCIP	Congenital disorders	RH13	Repair of club foot	
136	Referral hospital	Health services	Surgery	RH15	Repair of anorectal malformations and Hirschsprung's disease	
137	Referral hospital	Health services	Surgery	RH16	Repair of obstetric fistula	
138	Referral hospital	Health services	Surgery	RH17	Ventriculoperitoneal shunt	
139	Referral hospital	Health services	Surgery	RH18	Surgery for trachomatous trichiasis	
140	Population			P1	Mass media messages concerning sexual and reproductive health and mental health for adolescents (also included in HIV and Mental health packages of services)	MH3
141	Population			P2	Mass media messages concerning healthy eating or physical activity (also included in CVD and Musculoskeletal packages of services)	NCD4
142	Population			C25	Education campaign for the prevention of gender-based violence	RMNCAH8
143	Population			P4	Mass media encouraging use of condoms, voluntary medical male circumcision and STI testing	
144	Population			P6	Sustained integrated vector management for effective control of visceral Leishmaniasis, dengue, chikungunya, CCHF, and other nationally important causes of non-malarial fever vector borne NTDs	
145	Population			P13	Mass media messages concerning awareness on handwashing and health effects of household air pollution	
146	Population			P7	Conduct a comprehensive assessment of International Health Regulations (IHR) competencies using the Joint External Evaluation (JEE) tool	
147	Population			P10	Develop and implement a plan to ensure surge capacity in hospital beds, stockpiles of disinfectants, equipment for supportive care and personal protective equipment	
148	Population			P11	Develop plans and legal authority for curtaining interactions between infected persons and un-infected population and implement and evaluate infection control measures in health facilities	
149	Population			P8	Conduct simulation exercises and health worker training for outbreak events including outbreak investigation, contact tracing and emergency response	CD1
150	Population			P9	Decentralize stocks of antiviral medications to reach at risk groups and disadvantaged populations	
151	Population			P3	Mass media messages concerning use of tobacco (also included in CVD package of services)	

DCP: Disease Control Priorities; RPs: Research Priorities; CD: Communicable Diseases; NCD: Non-Communicable Diseases and Injuries; RMNCAH: Reproductive, Maternal, Newborn, Child, and Adolescent Health; MH: Mental Health; HSS: Health Systems and Services
